# The Intergeneration Long-Lasting Consequences of Pre-Conceptional Exposure to Sofosbuvir on the Ovarian Tissues of F1 Offspring: Experimental Study on Rats

**DOI:** 10.3390/ijms241813675

**Published:** 2023-09-05

**Authors:** Hala A. Hafez, Shimaa A. Mahmoud, Jehad F. Alhmoud, Rana H.M. Khafaga, Maher A. Kamel, Sara A. Shaker

**Affiliations:** 1 Department of Biochemistry, Medical Research Institute, Alexandria University, Alexandria 21561, Egypt; shimaa.abdelraheem@alexu.edu.eg (S.A.M.); maher.kamel@alexu.edu.eg (M.A.K.); sarah.a.shker@alexu.edu.eg (S.A.S.); 2 Department of Medical Laboratory Sciences, Jordan University of Science and Technology, Irbid 22110, Jordan; jalhmoud@just.edu.jo

**Keywords:** sofosbuvir, ovarian function, mitochondrial biogenesis and function, mtDNA

## Abstract

Sofosbuvir (SOF), a nucleos(t)ide polymerase inhibitor, has been used during the past decade for mass treatment of viral hepatitis C in endemic countries like Egypt, increasing the exposure of women at childbearing age to SOF. This study investigated the long-lasting consequences of the pre-conceptional exposure of young female rats to SOF on the ovarian tissues of F1 offspring and explored the possible molecular mechanisms of these intergenerational effects at various levels. The study was conducted on young female rats that were divided into control group and SOF-exposed group at a dose of 4 mg/kg/day for three months. After that, pregnancy was induced in both groups by mating with healthy male rats. After delivery, the female neonates were followed for 4 months, and the ovarian tissues were collected to assess the studied parameters. Pre-conceptional exposure to SOF affected the ovarian functions of F1 offspring through modulation of estrogen receptors, ovarian Kiss1 and its receptor, increased lipid peroxidation marker, DNA oxidation marker, and redox-sensitive nuclear factor kappa B, and decreased nuclear erythroid-2-related factor 2, mitochondrial function, and biogenesis. SOF affected the ovarian function of the F1 offspring by inducing oxidative stress and inflammation, leading to the modulation of mitochondrial functions and biogenesis.

## 1. Introduction

Hepatitis C virus (HCV) infection is an endemic threat to worldwide health. Egypt is one of the most HCV-affected countries in the world. The global health goal is to eliminate HCV infection by 2030; Egypt responded to that goal and initiated a nationwide initiative to screen and treat HCV-infected patients. This initiative is considered the largest public health effort to date in low and middle-income countries, increasing the total number of patients on treatment to more than 2 million [1]. The national treatment protocols used in this initiative include a sofosbuvir (SOF)-based regimen with or without other medications [2]. In 2014, SOF was approved for treating HCV as it cures more than 90% of patients and is effective against several HCV strains. SOF is a nucleotide analog, a phosphoramidate ester, and an organofluorine compound. It is functionally related to uridine 5′-monophosphate. SOF acts like other directly acting antivirals (DAAs) by targeting nonstructural 5B protein (NS5B) which is essential for viral RNA replication [3].

Mitochondrial dysfunction has been reported as an off-target effect of most DAA agents [4] through inhibiting mitochondrial DNA polymerase γ encoded by the POLG gene, thus halting mitochondrial DNA (mtDNA) replication which leads to mtDNA depletion, increased reactive oxygen species (ROS) production, and decreased synthesis of electron transport chain (ETC) proteins [5].

Mitochondria, “powerhouses of the cell,” are responsible for the production of most of the cell’s adenosine triphosphate (ATP) “energy currency”. Peroxisome proliferator-activated receptor gamma coactivator-1alpha (PGC-1α) is the master regulator of mitochondrial biogenesis [6]. The binding of PGC-1α with nuclear respiratory factor 1 (NRF1) induces the expression of mitochondrial transcription factor A (TFAM), which is a key regulator of mitochondrial DNA (mtDNA) transcription. The mtDNA encodes 13 proteins essential for ATP production by oxidative phosphorylation. NADH dehydrogenase is the largest first protein complex of the respiratory chains. This enzyme is essential for the normal functioning of cells, and mutations in its subunits lead to a wide range of inherited neuromuscular and metabolic disorders [7]. Citrate synthase (CS) is a nuclear-encoded mitochondrial enzyme that catalyzes the synthesis of citrate, the first step of the tricarboxylic acid cycle (TCA). It is found in the mitochondrial matrix of virtually all eukaryotic cells, where it is the rate-limiting enzyme of the TCA [8]. Mitochondrial malfunction leads to impaired ATP production and excessive ROS generation, which eventually leads to organ failure [9]. Nuclear factor erythroid-2-related factor 2 (Nfe2l2), the master regulator of the antioxidant response, stimulates the transcription of cytoprotective genes through binding to promoter sequences that involve conserved antioxidant response elements (AREs). This boosts levels of antioxidant enzymes and proteins that promote mitochondrial biogenesis, guaranteeing the replacement of damaged organelles [10].

Estrogen is another regulator of mitochondrial function and biogenesis through its alpha (ERα) and beta (ERβ) receptors, which can directly modulate the expression of genes NRFs and PGC-1 α [11]. Also, mtDNA contains hormone response elements, which allow the binding of estrogen receptors [12]. So, there is a close crosstalk between estrogen signaling and mitochondria in the ovarian tissues. Also, ovarian Kisspeptin, encoded by the Kiss1 gene, and its Kiss1 receptor (Kiss1R) have numerous potential roles in ovarian function, estrogen sensitivity, and female fertility. Recent studies identified Kiss1 as one of the differentially expressed genes regulated by ERα and ERβ [13,14].

Limited studies have explored the effects of SOF on the reproductive organs, which are very sensitive to chemicals due to the high multiplication rate of germ cells. However, the transmissible damage from one generation to another takes place mainly through this system and the intrauterine environment can influence the long-term health of offspring in a variety of ways [15]. Even though SOF shows a low overall toxicity profile, the in vivo effects of SOF are not fully understood, and the long-lasting intergenerational consequences have been subject to limited study. Furthermore, mitochondrial toxicity is one possible mechanism of nucleoside analog toxicity [16]. Our previous studies indicated that pre-conceptional SOF exposure in young female rats affected mitochondrial biogenesis and function in the ovaries, among other tissues, of the young females and their fetuses [17].

This study aimed to investigate the long-lasting consequences of the pre-conceptional exposure of young female rats to SOF in terms of the effects on the ovarian tissues of F1 offspring and to explore the possible molecular mechanisms of these intergenerational effects at various levels, including mitochondrial function and biogenesis, oxidative stress, inflammation, and the ovarian estrogen pathway.

## 2. Results

### 2.1. Ovarian Mitochondrial Parameters

**Figure 1:**
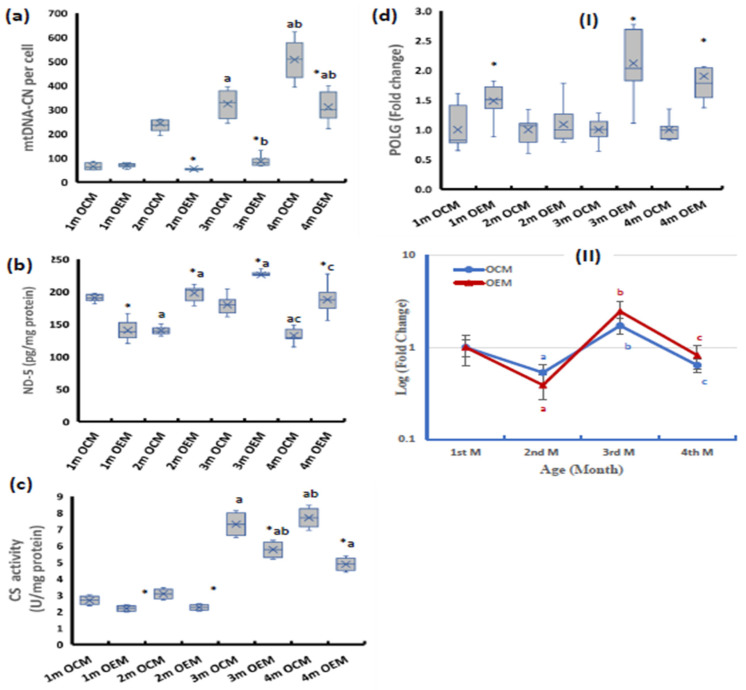
Ovarian levels of (**a**) mitochondrial DNA copy number (mtDNA-CN) per cell, (**b**) NADH dehydrogenase subunit-5 (ND-5) contents, (**c**) citrate synthase (CS) activity, (**d**) polymerase gamma (POLG) expression at (**I**) mRNA level. (**II**) Age-dependent change in mRNA expression relative to the 1st month. OCM: offspring of control mothers, OEM: offspring of SOF-exposed mothers, m: month. n = 8 in each subgroup, data presented as median (IQR). *: OEM significantly differs from OCM by Mann–Whitney test (*p* ≤ 0.05). a: significantly different from 1st month by Kruskal–Wallis test (*p* ≤ 0.05). b: significantly different from 2nd month by Kruskal–Wallis test (*p* ≤ 0.05). c: significantly different from 3rd month by Kruskal–Wallis test (*p* ≤ 0.05).

At the age of 1 month, the offspring of SOF-exposed mothers (OEMs) showed significantly lower NADH dehydrogenase subunit-5 (ND-5) and no significant change in mtDNA copy number (mtDNA-CN) from the offspring of control mothers (OCMs). At older ages (2, 3, and 4 months), the OEMs showed significantly lower mtDNA-CN and higher ND-5 contents than the OCMs (Figure 1a,b). In contrast, the CS activity in OEMs was significantly lower compared with the OCMs throughout the four-month follow-up period (Figure 1c). The POLG mRNA expression in the OEMs was significantly upregulated at the ages of 1, 3, and 4 months compared to the OCMs (Figure 1(dI)). 

Both groups of female offspring showed similar age-dependent patterns of change in mtDNA-CN in the ovarian tissues throughout the four-month follow-up period compared with the first month. Both groups showed an age-dependent increase in the mtDNA-CN through the four-month follow-up period (Figure 1a). In the OCMs, the ovarian ND-5 showed a significant decline in the second month compared with the first month then increased in the third month, and then significantly declined again in the fourth month. On the other hand, the OEMs showed a significant age-dependent increase in the ovarian ND-5 during the first three months of age then significantly declined at the age of four months (Figure 1b). Both groups showed nearly constant CS activity during the first 2 months of age with a marked increase in the 3rd and 4th months (Figure 1c). The POLG expression showed a significant decline at the age of 2 months then markedly increased at age 3 months to be higher than the baseline value at the age of 1 month. After that, at the age of 4 months, both groups of the offspring showed a significant decline in the expression of POLG to reach the baseline value again (Figure 1(dII)). 

### 2.2. Parameters of Mitochondrial Biogenesis Pathway

**Figure 2:**
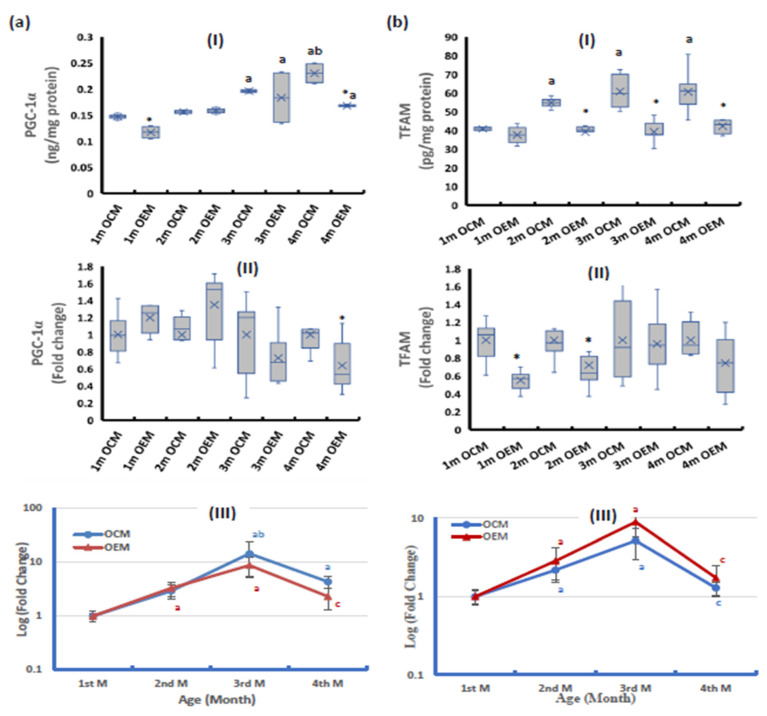
Ovarian expression of (**a**) peroxisome proliferator-activated receptor gamma coactivator-1alpha (PGC-1α) and (**b**) mitochondrial transcription factor A (TFAM) at (**I**) protein level and (**II**) mRNA level. (**III**) Age-dependent change in mRNA expression relative to the 1st month. OCM: offspring of control mothers, OEM: offspring of SOF-exposed mothers, m: month. n = 8 in each subgroup, data presented as median (IQR). *: OEM significantly differs from OCM by Mann-Whitney test (*p* ≤ 0.05). a: significantly different from 1st month by Kruskal–Wallis test (*p* ≤ 0.05). b: significantly different from 2nd month by Kruskal–Wallis test (*p* ≤ 0.05). c: significantly different from 3rd month by Kruskal–Wallis test (*p* ≤ 0.05).

The ovarian tissues of OEMs had significantly lower PGC-1α protein levels than the OCMs at the ages of 1 and 4 months (Figure 2(aI)), while the PGC-1α expression at mRNA level was significantly lower in OEMs at the age of 4 months (Figure 2(aII)).

The ovarian tissues of OEMs had significantly lower TFAM protein compared with the OCMs, from the 2nd month of age and thereafter (Figure 2(bI)). At the mRNA level, the OEMs had significantly downregulated TFAM expression during the first 2 months of age after which no significant difference was observed compared with the OCMs (Figure 2(bII)). 

During the 4-month follow-up period, the OCMs showed an age-dependent increase of PGC-1α protein especially at ages 3 and 4 months, while the OEMs showed a nearly constant level during the first 2 months of age then mildly increasing at the age of 3 months with no further increase at age 4 months. At the mRNA level, both sets of offspring showed an age-dependent increase in PGC-1α during the first 3 months of age then the expression showed a decline, especially in the OEMs (Figure 2(aIII)). 

Regarding TFAM, the OCMs showed an age-dependent increase of TFAM protein, while the OEMs showed no change in TFAM protein during the follow-up period. At the mRNA level, both sets of offspring showed a significant age-dependent increase in TFAM during the first 3 months of age then the expression significantly declined to nearly the baseline values (Figure 2(bIII)). 

### 2.3. Redox Parameters

**Figure 3:**
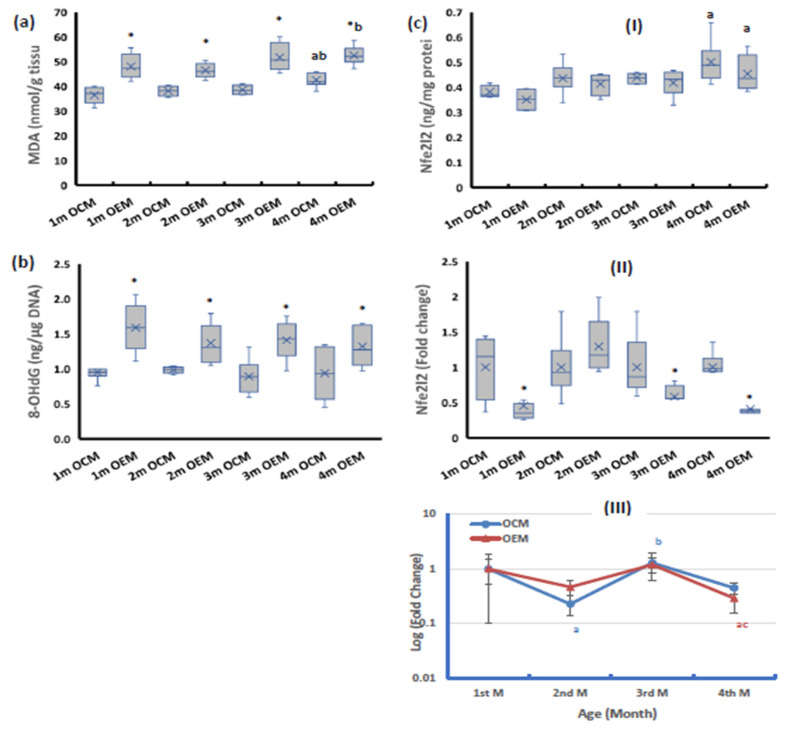
Ovarian levels of (**a**) malondialdehyde (MDA) (**b**) 8-Hydroxy deoxyguanosine (8-OHdG) and (**c**) nuclear factor erythroid-2-related factor 2 (Nfe2l2) expression at (**I**) protein level and (**II**) mRNA level. (**III**) Age-dependent change in mRNA expression relative to the 1st month. OCM: offspring of control mothers, OEM: offspring of SOF-exposed mothers, m: month. n = 8 in each subgroup, data presented as median (IQR). *: OEM significantly differs from OCM by Mann–Whitney test (*p* ≤ 0.05). a: significantly different from 1st month by Kruskal–Wallis test (*p* ≤ 0.05). b: significantly different from 2nd month by Kruskal–Wallis test (*p* ≤ 0.05). c: significantly different from 3rd month by Kruskal–Wallis test (*p* ≤ 0.05).

Both markers of ovarian oxidative stress (malondialdehyde (MDA) and 8-hydroxy deoxyguanosine (8-OHdG)) were significantly higher in the OEM group compared with the OCM group throughout the 4-month follow-up period. In both groups, OCM and OEM, the MDA and 8-OHdG contents appeared to be constant during the first 3 months of age then the MDA significantly increased at the 4th month of age, whereas the 8-OHdG contents remained constant through the 4-month follow-up period (Figure 3a,b). 

The ovarian protein level of Nfe2l2 showed no significant change between the studied group throughout the study period (Figure 3(cI)). At the mRNA level, the OEMs showed significant downregulation of Nfe2l2 expression compared with the OCM group at all studied ages except at the age of 2 months (Figure 3(cII)). During the 4-month follow-up period, the OCMs and OEMs showed constant protein contents of Nfe2l2 during the first 3 months of age, after which its levels significantly increased compared with the baseline value. At the mRNA level, both sets of offspring showed significant fluctuation of Nfe2l2 expression, significantly declining at the 2nd month of age followed by significant upregulation to the baseline value in the 3rd month and then downregulation again at the age of 4 months (Figure 3(cIII)).

### 2.4. The Ovarian Expression of Nuclear Factor kappa-B (NF-κB) 

**Figure 4:**
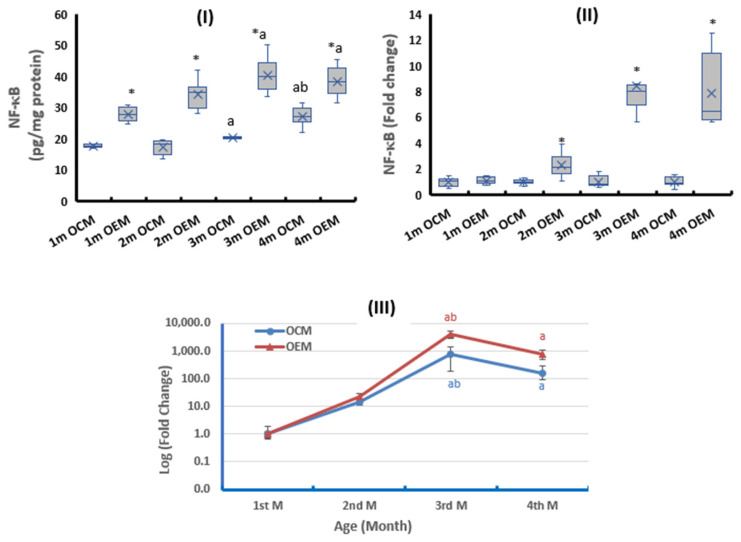
Ovarian expression of nuclear factor kappa-B (NF-κB) at (**I**) protein level and (**II**) mRNA level. (**III**) Age-dependent change in mRNA expression relative to the 1st month. OCM: offspring of control mothers, OEM: offspring of SOF-exposed mothers, m: month. n = 8 in each subgroup, data presented as median (IQR). *: OEM significantly differs from OCM by Mann–Whitney test (*p* ≤ 0.05). a: significantly different from 1st month by Kruskal–Wallis test (*p* ≤ 0.05). b: significantly different from 2nd month by Kruskal–Wallis test (*p* ≤ 0.05).

The ovarian NF-κB protein levels were significantly higher in OEMs compared with the OCMs at all studied ages (Figure 4I). At the mRNA level, the OEMs group showed no significant difference in NF-κB expression compared with the OCMs at the age of 1 month, after which the expression was markedly induced compared with the OCMs (Figure 4II). The NF-κB expression in the ovarian tissues the protein level showed nearly constant levels during the first two months, then significantly increased during the next two months in both groups, especially in the OEMs. At the mRNA level, the expression of NF-κB in both groups showed an age-dependent exponential increase during the first three months of age then the expression declined in the 4th month (Figure 4III).

### 2.5. Ovarian Expression of the Components of the Estrogen Pathway

**Figure 5:**
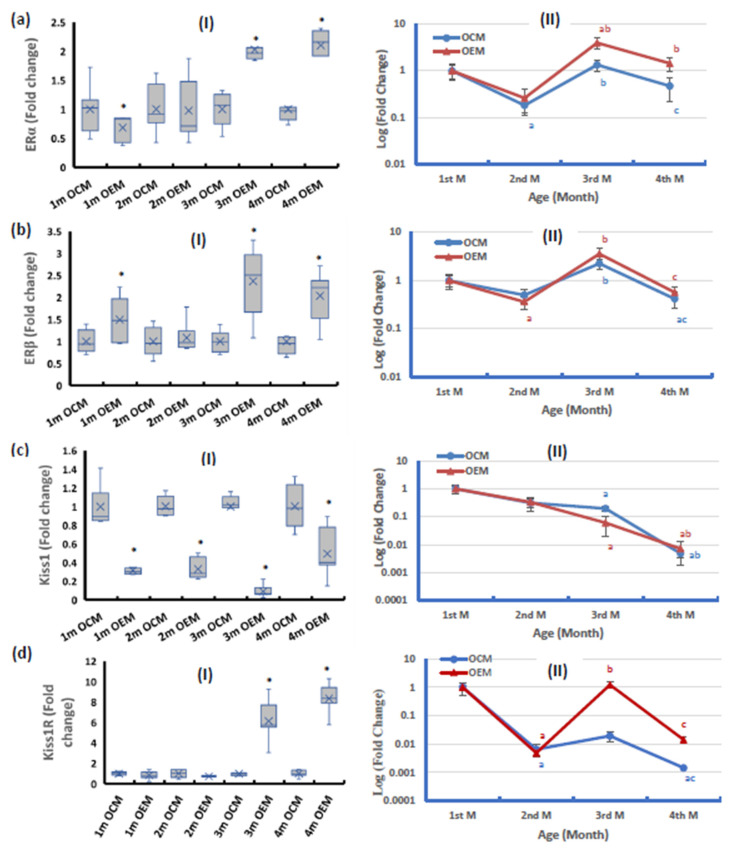
Ovarian expression of (**a**) estrogen receptor alpha (ERα), (**b**) estrogen receptor beta (ERβ), (**c**) Kiss1, and (**d**) Kiss1 receptor (Kiss1R) at (**I**) mRNA level. (**II**) Age-dependent change in mRNA expression relative to the 1st month. OCM: offspring of control mothers, OEM: offspring of SOF-exposed mothers, m: month. n = 8 in each subgroup, data presented as median (IQR). *: OEM significantly differs from OCM by Mann–Whitney test (*p* ≤ 0.05). a: significantly differ from 1st month by Kruskal–Wallis test (*p* ≤ 0.05). b: significantly differ from 2nd month by Kruskal–Wallis test (*p* ≤ 0.05). c: significantly differ from 3rd month by Kruskal–Wallis test (*p* ≤ 0.05).

The ovarian ERα expression showed significant downregulation in the OEMs compared with the OCM group at the age of one month. While there was no significant change at the age of 2 months, the expression was significantly upregulated in OEMs compared with the OCMs from the 3rd month of age and thereafter (Figure 5(aI)). On the other hand, the OEMs showed significant upregulation in ERβ mRNA expression compared with the OCMs at all ages except for the age of 2 months (Figure 5(bI)). OCMs and OEMs both showed a similar pattern of expression of ERα and ERβ in ovarian tissues during the 4-month follow-up period compared with the 1st month value (baseline). The expression showed a significant decline in the 2nd month of age, then significant upregulation to reach expression levels higher than the baseline value, and then declined again at the age of 4 months to about half the baseline expression. However, the amplitudes of ERα expression in the OEMs were higher at the 3rd and 4th months by about three-fold compared with the expression values in OCMs at the same ages (Figure 5(aII,bII)).

The ovarian Kiss1 mRNA expression was significantly downregulated in the OEMs compared with the OCM group at all studied ages (Figure 5(cI)). The pattern of change of Kiss1 mRNA expression in both OCMs and OEMs showed a stepwise age-dependent decline to reach the lowest expression level at 4 months old (Figure 5(cII)).

The ovarian Kiss1R mRNA expression in the OEM group showed no significant changes during the first 2 months of age compared with the OCMs, after which the expression was markedly upregulated at the ages of 3 and 4 months (Figure 5(dI)). The pattern of expression of Kiss1R in ovarian tissues of OCMs during the 4-month follow-up period compared with the 1st-month value showed a significant decline in the 2nd month and stayed constant to the 3rd month of age, and then the expression declined to the lowest level at the age of 4 months. The OEMs showed a different pattern of changes as the expression declined in the 2nd month of age then increased and returned to the base value in the 3rd month of age, then declined back (Figure 5(dII)).

### 2.6. Correlation Studies

The statistical analysis using Spearman’s rank correlation (Table 1) revealed that the ND-5 content was positively correlated with NF-κB content while negatively correlated with TFAM content and mtDNA-CN. The POLG mRNA expression was positively correlated with ND-5 content, 8-OHdG content, MDA content, and NF-κB content. The PGC-1α content was positively corrected with Nfe2l2 content, TFAM content, and mtDNA-CN. The TFAM content was positively correlated with mtDNA-CN while negatively correlated with NF-κB content. The MDA content was positively corrected with NF-κB content. The 8-OHdG was positively correlated with NF-κB content while negatively correlated with TFAM content and mtDNA-CN. The Nfe2l2 content was positively corrected with TFAM content and mtDNA-CN. The ERα mRNA expression was positively correlated with Kiss1R mRNA expression, ND-5 content, and NF-κB content. The ERβ mRNA expression was positively correlated with 8-OHdG, MDA content, and NF-κB content while negatively correlated with Kiss1 mRNA expression. The CS activity was positively correlated with the contents of PGC-1α, TFAM, Nfe2l2, and mtDNA-CN while negatively correlated with 8-OHdG. 

## 3. Discussion

Our recent study provided preliminary evidence for the detrimental effects of SOF on the pregnancy outcomes of exposed female rats [17], and in the present study, we extend our finding to the intergenerational detrimental effects of maternal SOF exposure on the ovarian tissues of the female rat postnatal offspring. The ovarian tissues of the F1 female offspring of mothers pre-conceptionally exposed to SOF showed an imbalance in mitochondrial homeostasis, which was associated with shifting in the redox status, oxidative DNA damage, inflammation, and modulation of estrogen receptors and Kiss1 expression and its receptor. 

Optimum oocyte development and competence require a time-dependent increase in mitochondrial functions and biogenesis [18]. In the present study, the OCMs showed competent oocyte development revealed as a time-dependent increase in the mtDNA-CN, which was associated with a similar age-dependent increase in the PGC-1α/TFAM pathway of mitochondrial biogenesis during the first three months. In line with our study, Perry et al. [19] suggested that mitochondrial activity is an indicator of the developmental competence of the oocyte. In the 4th month, PGC-1α/TFAM showed decreased mRNA expression without a decrease in their protein contents. This discrepancy reveals the existence of post-transcriptional/translational regulatory mechanisms that need further investigation but could be explained by the report of Miyamoto et al., [20] who proposed that repeated ovulation not only decreases the number of oocytes but also decreases the expression of TFAM. On the other hand, the ovarian tissues of the OEMs had lower mtDNA-CN compared with the control offspring; also, mtDNA-CN did not show normal age-dependent increase during the first 3 months of age, then showed a delayed increase at the 4th month. This abnormal pattern of mitochondrial biogenesis is associated with a significant decline in the PGC-1α/ TFAM pathway, which was confirmed by the correlation data indicating the positive association between the mtDNA-CN and the PGC-1α and TFAM. 

A study revealed that PGC-1α was downregulated in diminished ovarian reserve patients compared with normal ovarian reserve patients in the cumulus granulosa cells, which exist in proximity to the oocyte and are involved in the growth and maturation of oocytes [21]. Also, PGC-1α has an important role in the development of goat follicles [22]. The reduced TFAM expression may induce mitochondrial dysfunction and redox imbalance, which leads to disturbance of steroidogenesis in ovarian goat follicles [23]. TFAM is also known to be a direct regulator of mtDNA-CN [24], and TFAM gene allele deletion decreased mtDNA-CN in mouse oocytes which affected fertilization and embryo implantation [25]. 

At the functional level, the ovarian tissues of the OEMs had a significantly lower content of ND-5 compared with the control at the 1st month of age and then the ND-5 content significantly increased to become higher than the control offspring from the 2nd month of age and thereafter. NADH dehydrogenase, complex I, is considered the rate-limiting step in ETC. Mutations in complex I subunit genes have been associated with ETC disturbances and ROS production [26]. The contradictory results regarding the increased content of ND-5 and the decline of mtDNA-CN in the OEMs may be explained in the context of adaptation as the enhanced ND-5 content may compensate for mitochondrial biogenesis decline. Also, the increased DNA oxidative damage in the ovarian tissues may be mutagenic, especially in the mitochondria due to the close vicinity of mtDNA to the source of ROS, and ND-5 may be a target of this mutagenicity that results in unfunctional ND-5 that may impair the ETC and further increase ROS production. The correlation data support this suggestion as indicated by the negative correlation between ND-5 content and TFAM content and mtDNA-CN. 

Regarding CS activity, a biomarker for mitochondrial activity, in the present study, the OEMs showed lower CS activity at all studied ages. The CS activity was reduced in the ovarian tissues of a rat model of polycystic ovary syndrome (PCOS), revealing dysfunctional mitochondria and collapsed bioenergetics [27]. Also, CS downregulation induces a reduction in ATP production, an increase in oxidative stress and cell apoptosis [28]. Moreover, the induction of mitochondrial dysfunction increases oxidative stress with reduced CS activity, ATP production, and mitochondrial biogenesis [29]. In the present study, the CS activity is negatively associated with 8-OHdG and positively associated with NFe2l2 and mitochondrial biogenesis (PGC-1α, TFAM, and mtDNA-CN). Most DAA agents affect the mitochondria by inhibiting the POLG gene [4], leading to mtDNA depletion, increased ROS production, and decreased synthesis of ETC proteins [5]. In the present study, the ovarian tissues of OEMs had upregulated expression of the POLG gene as early as the 1st month of age and thereafter, which may be considered a compensatory mechanism to restore the mitochondrial functions and counteract the oxidative stress and inflammation as indicated by the positive association between the POLG expression and ND-5 content, 8-OHdG content, MDA, and NF-κB expression and negative association with Nfe2l2.

The ovarian tissues of the female offspring of SOF-exposed mothers suffered from oxidative stress and inflammation due to high lipid peroxidation markers (MDA) and DNA oxidative damage markers (8-OHdG), suppressed Nfe2l2 expression, and elevated NF-κB expression at mRNA and protein levels during the four months. The presence of oxidative stress in the ovarian tissue in the OEMs could affect the oocyte quality and hence reproduction. The absence of changes in the protein content of Nfe2l2 could be explained by the existence of post-transcriptional and/or post-translational regulatory pathways that need to be investigated. The knockdown of the Nfe2l2 gene impaired the ovarian function and antioxidant capacity in premature ovarian failure in mice [30], and the activation of its pathway alleviated the oxidative stress induced by cryopreservation of the ovarian tissue [31]. Also, Nfe2l2 cross-talks with the regulation of mitochondrial biogenesis by PGC-1α, as indicated in the present study by the positive association of PGC-1α content with Nfe2l2 content. Furthermore, the Nfe2l2 content is positively correlated with mtDNA-CN and TFAM. The knockout of PGC-1α irregulates the Nfe2l2-dependent mitochondrial biogenesis [32], the deficiency of Nfe2l2 impairs mitochondrial biogenesis, and the downregulation of PGC-1α inhibits the binding of Nfe2l2 to the antioxidant-responsive genes [33,34].

In the OEMs, the elevated oxidative stress resulted in the observed marked elevation of NF-κB expression that exaggerated the oxidative stress in a vicious cycle. Paciolla et al. [35] reported that the NF-κB pathway could have a crucial role in female reproduction. Mitochondria-derived ROS release mtDNA and directly induce the stimulation of innate immune responses, involving activation of the inflammasome and the NF-κB signaling pathways [36].

The observed changes in ovarian mitochondrial homeostasis, redox status, and inflammatory status in the offspring of SOF-exposed mothers were associated with a dysregulation in the components of estrogen signaling, including ERα/ERβ and Kiss1/Kiss1R. The ERs showed biphasic changes as the expression of ERα and ERβ was markedly downregulated at the 2nd month of age and then upregulated at the 3rd month of age and then downregulated again at the age of 4 months in both sets of offspring. The OEMs showed low expression of ERα at the age of 1 month and then the expression became higher at the age of 3 and 4 months. On the other hand, the expression of ERβ was significantly higher in the OEMs as early as the 1st month of age and thereafter. These patterns of changes may indicate abnormalities in the estrogen signaling pathways, and the enhanced expression of ERα/ERβ may be a compensatory mechanism for the possible decline in levels of estrogen, but this suggestion needs further study. Also, the OCMs showed a relative fluctuation of Kiss1/ Kiss1R expression as it showed downregulation in Kiss1 mRNA expression during the 4 months of the follow-up period and upregulation of its receptor (Kiss1R) at the older age points (3 and 4 months), indicating a detrimental effect on ovarian function. In line with the present study, Khristi et al. [37] reported that the loss of Kiss1 expression impaired the development and maturation of follicles and oocytes. On the other hand, Kiss1R expression showed a dramatic increase in the 3rd and 4th months of the follow-up period. This discrepancy between Kiss1 and Kiss1R expression is in line with Marcondes et al., [38] who reported that Kiss1 expression was downregulated in a rat model of PCOS, suggesting anovulation, whereas Kiss1R was upregulated which was probably a compensatory mechanism for Kiss1 downregulation. 

Ovarian estrogens are essential for female reproduction through the estrous cycle regulation. Estrogen exerts its effects by binding to ERs and its levels fluctuate during the estrous/menstrual cycle and produce definite effects on the female reproductive tract [39]. The estrogen/ER pathways are involved in the development and physiology of female organs such as mammary glands, uterus, and vagina [40]. Also, estrogen/ER pathways directly modulate mitochondrial biogenesis and function [11]. The ovarian Kiss1/ Kiss1R system performs numerous functions in the ovary at distinct physiological stages involving follicular development, steroidogenesis, oocyte maturation, and ovulation [14,41]. The expression patterns of Kiss1/ Kiss1R can be strongly impacted by differences in the age of the ovarian tissues and cells acquired from various estrous/menstrual cycles [14]. Similarly, most of the measured parameters may be cycling with estrogen levels due to the estrous cycle, so sampling during the exact phase of the estrous cycle will give a clear image of the above-mentioned fluctuations, which was the main limitation in our study. However, the correlation results may indicate possible crosstalk between the components of the estrogen signaling pathway (ERs/Kiss1/Kiss1R), oxidative stress, and mitochondrial biogenesis, but such a link needs further investigation.

According to the present data, we can order the abnormalities in the ovarian tissues as follows: the enhanced oxidative stress and inflammation start as early as the 1st month of age and precede the other changes; these abnormalities may drive the other changes in ovarian tissues that start later from the 2nd month of age, including impaired mitochondrial biogenesis, and estrogen signaling. For most studied parameters, it is clear that marked changes occur after the 2nd month of age which coincide with the age of puberty onset and menstruation in the female rat offspring of healthy or SOF-exposed mothers. 

## 4. Materials and Methods

### 4.1. Experimental Animals

The study was conducted on 16 female Wistar albino rats about 2 months old, weighing 115–125 g and purchased from the animal house of the Medical Research Institute, Alexandria, Egypt. All rats had free access to food and water with a 12:12 h light/dark cycle and constant environmental conditions before experimentation and thereafter. 

### 4.2. Ethical Statement

All procedures were performed in accordance with the Institutional Animal Care And Use Committee (IACUC), Alexandria University, Egypt (Approval number: AU0122142421). All experiments fulfilled the guidelines of the National Institutes of Health guide for the care and use of laboratory animals (NIH Publications No. 8023, revised 1978) and the recommendations of Egypt’s guide for the care and use of laboratory animals. All efforts were made to curb the distress of rats during the experimental period.

### 4.3. Drug

Sofosbuvir ((SOF), a product of Pharco Pharmaceuticals, Alexandria, Egypt), was available in the form of tablets with the trade name ‘Gratisovir’. Each tablet contains 400 mg of sofosbuvir; these tablets were dissolved in distilled water and given to rats orally by gastric tube at a dose of 4 mg/kg per day for 3 months [17,42].

### 4.4. Experimental Design

The rats were randomly allocated using block randomization into two groups; Group I (Control group) included 8 healthy female rats that were maintained under a normal diet and received a placebo (distilled water orally by gastric tube) for three months, and Group II (SOF-exposed group): included 8 healthy female rats that were supplemented with 4 mg/kg/day of the SOF drug for three months. After the treatment period, Pregnancy was induced in control and exposed females by mating with healthy male rats overnight. After delivery, the female neonates (OCMs and OEMs) were followed and 8 offspring from each group were sacrificed every month for 4 months.

### 4.5. Tissue Preparation

After the experimental period, rats were sacrificed under deep anesthesia using isoflurane (100%) to obtain ovarian tissues. The excised ovarian tissues were rinsed with saline and then divided into three aliquots: one used for total RNA extraction for quantitative real-time polymerase chain reaction (qPCR), the second aliquot used for total DNA extraction for assessment of mtDNA-CN, and 8-OHdG, and the third aliquot homogenized in phosphate buffer saline (PBS) pH 7.4 in the ratio of 1:9, the homogenate used for determination of MDA levels, then centrifuged at 10,000 rpm, at 4 °C for 20 min and the supernatants were stored at −20 °C for subsequent determination of total protein, CS activity, ND-5, PGC-1α, TFAM, Nfe2l2 and NF-κB contents. 

### 4.6. Determination of Malondialdehyde as a Marker for Lipid Peroxidation 

Malondialdehyde (MDA) in the whole homogenate was determined according to the method of Draper and Hadley [43]. The tissue samples are heated with thiobarbituric acid (TBA) at low pH. The resulting pink chromogen has a maximal absorbance of 532 nm. Results were expressed as nmol MDA/g tissue by dividing the concentration of MDA in the sample by the g tissue in the same sample.

### 4.7. Elisa Measurements

Determination of ND-5, PGC-1α, TFAM, Nfe2l2, and NF-κB contents in the ovarian tissues were assayed using the following rat ELISA kits according to the manufacturer’s instructions; ND-5 (Chongqing Biospes Co., Ltd., Chongqing, China, Cat no. BYEK3461), PGC-1α (MyBioSource, Inc., San Diego, CA, USA Cat no. MBS2706379), TFAM (MyBioSource, Inc., USA, Cat no. MBS1600609), Nfe2l2 (Chongqing Biospes Co., Ltd., Cat no. BYEK2318), and NF-κB (Chongqing Biospes Co., Ltd., Cat no. BYEK3040).

### 4.8. Ovarian Citrate Synthase (CS) Activity

Citrate synthase (CS) activity was measured through the formation of the -SH group released from CoA-SH by use of the reactive Ellman reagent (5,5′-dithiobis [2-nitrobenzoic], DTNB) and monitoring the absorbance at 412 nm [44].

### 4.9. Determination of Total Protein

A modification method of Lowry et al. was used for the determination of protein in the samples [45].

### 4.10. Gene Expression Analysis Using Quantitative Real-Time Polymerase Chain Reaction (qPCR)

Quantitative analysis of PGC-1α, NRF1, TFAM, POLG, Nfe2l2, NF-κB, ERα, ERβ, Kiss1, and Kiss1R expression in ovarian tissue was performed using two-step reverse transcriptase-PCR. The mRNA was isolated from the ovarian tissues using miRNeasy Mini Kit (Qiagen, Hilden, Germany, catalog no. 217004) according to the manufacturer’s instructions. The reverse transcription was done using TOPscript™ RT DryMIX (dT18/dN6 plus) kit according to the manufacturer’s instructions (Enzynomics Co., Ltd., Daejeon, Republic of Korea, cat number RT220). Then, Relative quantifications were performed using ViPrime PLUS Taq qPCR Green Master Mix I (Vivantis Technologies, Selangor Darul Ehsan Malaysia, cat. Number QLMM12) and using the specific primer sets for each gene (Table 2). Quantitative PCR amplification conditions were adjusted as an initial denaturation at 95 °C for 5 min and then 45 cycles of PCR for amplification as follows: Denaturation at 94 °C for 20 s, annealing at 55 °C for 20 s and extension at 70 °C for 15 s [46].

Data was collected using Bio-Rad CFX Maestro version 2.3 (Bio-Rad, Inc., Hercules, CA, USA). The relative expression of PGC-1α, NRF1, TFAM, POLG, Nfe2l2, NF-κB, ERα, ERβ, Kiss1, and Kiss1R was quantified relative to the expression of the reference gene (18S rRNA) in the same sample by calculating and normalizing the threshold cycles (Ct) values of the target to that of 18S rRNA. The fold change of expression of each gene at each age in OEM was calculated relative to the OCMs using the 2^−ΔΔCt^ method, while the age-dependent change of the expression of each gene in OCMs and OEMs was calculated relative to its expression at the first month of age using the livak 2^−ΔΔCt^ method [47].

### 4.11. Determination of Mitochondrial DNA Copy Number per Cell

A real-time PCR assay was used to assay the mtDNA relative to nuclear DNA. After total genomic DNA isolation, we used a specific primer pair for mtDNA sequence (mtDNA) and a primer pair specific for the nuclear PGC-1α (nPGC-1α) gene to perform the same number of PCR cycles and calculate the relative mtDNA signal to nuclear DNA signal [46]. The nuclear gene was used to quantify nuclear DNA (nDNA) and, therefore, normalization of the mtDNA amount per the nDNA of the diploid cells used the equation:

R = 2^−ΔCt^ where ΔCt = Ct_mtDNA_ − Ct_nuclear_

The total DNA was isolated from the different tissues using a DNeasy kit (Hilden, Germany) according to the manufacturer’s instructions. The primer pair used for mtDNA (NC_040919.1) was Forward: AATGGTTCGTTTGTTCAACGATT and Reverse: AGAAACCGACCTGGATTGCTC; the primer pair for the nuclear PGC-1α gene (NM_031347.1) was Forward: ATGAATGCAGCGGTCTTAGC and Reverse: AACAATGGCAGGGTTTGTTC. PCR reactions were carried out using the master mix SsoAdvanced Universal SYBR Green Supermix (Bio-Rad, Inc. USA, catalog number 1725270) under the following conditions: 98 °C for 2 min followed by 45 cycles of 98 °C for 10 s and 55 °C for 30 s.

### 4.12. Determination of 8-Hydroxy Deoxyguanosine (8-OHdG)

The isolated DNA samples from the ovarian tissues were enzymatically digested as described previously [17] and used to assess the content of 8-OHdG using an ELISA kit (Chongqing Biospes Co., Ltd., Catalog number BYEK3439) according to the manufacturer’s instructions.

### 4.13. Statistical Analysis

Data were analyzed using SPSS software package version 18.0 (SPSS, Chicago, IL, USA). The data were expressed as median (interquartile range (IQR)) and analyzed using the Mann–Whitney test to compare between different groups in the same month, while comparisons between the follow-up period in the same group were made by Kruskal–Wallis one-way analysis of variance according to the test of normality. Spearman’s rank was used for the correlation study. The *p*-value was assumed to be significant at *p* ≤ 0.05 [48]. The pattern of change in the mRNA expression was calculated using the fold difference method at different time points relative to the 1st month as a baseline value.

## 5. Conclusions

The present study is the first, according to our knowledge, that demonstrates the long-term determinantal effects of SOF on the female OEMs. The mechanism(s) of these intergeneration effects is(are) unclear and intensive study is needed. However, we can suggest a different possible mechanism that may participate: (1) SOF may directly or indirectly affect the quality of the maternal ova by disrupting the regulation of mitochondrial machinery that drives offspring abnormalities developed later after pregnancy, (2) SOF may induce oxidative damage and placental abnormalities early in pregnancy that may affect the nutrient supply to the fetus which may result in fetal programming for long-term consequences after birth leading to mitochondrial dysfunction, and (3) SOF may affect hormonal homeostasis pre-conception, during pregnancy, and after birth, which may affect ovarian development. The extent and the interplay of these possible mechanisms need further investigation.

## Figures and Tables

**Table 1:** Correlation studies in the ovarian tissue (Spearman’s rank correlation “r”).

	ND-5 Protein	POLG mRNA	PGC-1α Protein	TFAM Protein	MDA	8-OHdG	mtDNA-CN	ERα mRNA	ERβ mRNA
ND-5 protein	−	0.338 *	−0.006	−0.406 *	0.258 *	0.218	−0.393 *	0.422 *	0.296 *
TFAM protein	−0.406 *	−0.282 *	0.510 *	−	−0.419 *	−0.439 *	0.685 *	−0.038	−0.349 *
MDA	0.258 *	0.519 *	−0.045	−0.419 *	−	0.615 *	−0.079	0.372 *	0.623 *
8-OHdG	0.218	0.413 *	−0.288 *	−0.439 *	0.615 *	−	−0.294 *	0.138	0.556 *
Nfe2l2 protein	−0.066	0.055	0.536 *	0.423 *	0.074	−0.204	0.490 *	0.096	0.036
NF-κB protein	0.373 *	0.542 *	0.208	−0.420 *	0.833 *	0.545 *	−0.025	0.400 *	0.533 *
mtDNA-CN	−0.393 *	0.015	0.636 *	0.685 *	−0.079	−0.294 *	−	0.170	−0.034
Kiss1 mRNA	−0.484 *	−0.521 *	0.139	0.593 *	−0.571 *	−0.610 *	0.420 *	−0.221	−0.510 *
Kiss1R mRNA	0.284 *	0.371 *	0.244	−0.103	0.355 *	0.079	0.285 *	0.559 *	0.323 *
CS activity	−0.049	0.038	0.789 *	0.588 *	−0.093	−0.327 *	0.766 *	0.309 *	−0.039

* Statistically significant at *p* ≤ 0.05. ND-5: NADH dehydrogenase subunit-5, POLG: polymerase gamma, PGC-1α: peroxisome proliferator-activated receptor gamma coactivator-1alpha, TFAM: mitochondrial transcription factor A, MDA: malondialdehyde, 8-OHdG: 8-Hydroxy deoxyguanosine, mtDNA-CN: mitochondrial DNA copy number, Nfe2l2: nuclear factor erythroid-2-related factor 2, ERα: estrogen receptor alpha, ERβ: estrogen receptor beta, Kiss1R: Kiss1 receptor, NF-κB: nuclear factor kappa-B, CS: citrate synthase.

**Table 2:** Primers used for real-time PCR.

Gene	Accession Number	Primer Sequence	
*18S rRNA* (Reference gene)	NR_046237.2	F:	GTAACCCGTTGAACCCCATT
		R:	CAAGCTTATGACCCGCACTT
*PGC-1α*	NM_031347.1	F:	GTGCAGCCAAGACTCTGTATGG
		R:	GTCCAGGTCATTCACATCAAGTTC
*TFAM*	NM_031326.2	F:	CCCACAGAGAACAGAAACAG
		R:	CCCTGGAAGCTTTCAGATACG
*POLG*	NM_053528.1	F:	GGACCTCCCTTAGAGAGGGA
		R:	AGCATGCCAGCCAGAGTCACT
*Nfe2l2*	NM_017008.4	F:	CGAGATATACGCAGCAGGAGAGGTAAG
		R:	GCTCGACAATGTTCTCCAGCTT
NF-κB (P65)	NM_199267.2	F:	CAGGACCAGGAACAGTTCGAA
		R:	CCAGGTTCTGGAAGCTATGGAT
*ERα*	NM_012689.1	F:	ATGAGAGCTGCCAACCTT
		R:	AACAAGGCACTGACCATC
*ERβ*	NM_012754	F:	AGGTGCTAATGGTGGGACTG
		R:	ACTTTCTGCCTCCTGGTTTG
*Kiss1*	NM_181692.1	F:	GCTGCTGCTTCTCCTCTGTGT
		R:	CTGTTGGCCTGTGGGTTCA
*Kiss1R*	NM_023992.2	F:	AGCACATGCAGACCGTCACC
		R:	GACGAATTTGCACATGAAGTCTCC

F: Forward, R: Reverse. *PGC-1α*: Peroxisome proliferator-activated receptor gamma coactivator-1alpha, *TFAM*: mitochondrial transcription factor A, *POLG*: polymerase gamma, *Nfe2l2*: nuclear factor erythroid-2-related factor 2, NF-κB: nuclear factor kappa-B, *ERα*: estrogen receptor alpha, *ERβ*: estrogen receptor beta, *Kiss1R*: Kiss1 receptor.

## Data Availability

The data are available upon request.
